# Veno-Arterial Extracorporeal Membrane Oxygenation for Patients Undergoing Heart Transplantation: A 7-Year Experience

**DOI:** 10.3389/fmed.2021.774644

**Published:** 2021-12-16

**Authors:** Jun-yi Hou, Xin Li, Shou-guo Yang, Ji-li Zheng, Jie-fei Ma, Ying Su, Yi-jie Zhang, Ke-fang Guo, Guo-wei Tu, Zhe Luo

**Affiliations:** ^1^Department of Critical Care Medicine, Zhongshan Hospital, Fudan University, Shanghai, China; ^2^Department of Cardiovascular Surgery, Zhongshan Hospital, Fudan University, Shanghai, China; ^3^Department of Nursing, Zhongshan Hospital, Fudan University, Shanghai, China; ^4^Department of Critical Care Medicine, Xiamen Branch, Zhongshan Hospital, Fudan University, Xiamen, China; ^5^Department of Anesthesiology, Zhongshan Hospital, Fudan University, Shanghai, China; ^6^Shanghai Key Lab of Pulmonary Inflammation and Injury, Fudan University, Shanghai, China

**Keywords:** heart failure, heart transplantation, primary graft dysfunction, veno-arterial extracorporeal membrane oxygenation, cardiogenic shock

## Abstract

**Objective:** Primary graft dysfunction (PGD) is the leading cause of early death after heart transplantation. Veno-arterial extracorporeal membrane oxygenation (VA-ECMO) can provide temporary mechanical circulatory support and time for functional recovery of the transplanted heart. The purpose of this study was to analyze the timing and prognoses of VA-ECMO in patients with severe PGD after heart transplantation.

**Methods:** A total of 130 patients underwent heart transplantation at the Zhongshan Hospital Affiliated with Fudan University between January 2014 and December 2020. All patients received basiliximab immunoinduction and a classic double vena cava anastomosis orthotopic heart transplantation. Among them, 29 patients (22.3%) developed severe PGD in the early postoperative period. VA-ECMO was performed in patients with difficulty weaning from cardiopulmonary bypass (CPB) or postoperative refractory cardiogenic shock. Patients were divided into two groups according to whether or not they were successfully weaned from VA-ECMO (patients who survived for 48 h after weaning and did not need VA-ECMO assistance again). The perioperative clinical data were recorded, and all patients were followed up until discharge. Early outcomes were compared between groups.

**Results:** A total of 29 patients with VA-ECMO support after heart transplantation were included in this study. The proportion of patients receiving VA-ECMO was 22.3% (29/130). Nineteen patients (65.5%) needed VA-ECMO due to difficulty with weaning from CPB, and 10 patients required VA-ECMO for postoperative cardiogenic shock. Nineteen patients (65.5%) were successfully weaned from VA-ECMO. Overall, in-hospital mortality of VA-ECMO support patients was 55.2%. The main causes of death were ventricular fibrillation (four cases), major bleeding (three cases), infection (four cases), and graft failure (five cases).

**Conclusion:** Despite advances in heart transplantation, severe PGD remains a lethal complication after heart transplantation. At present, the treatment for severe PGD after heart transplantation is a challenge. VA-ECMO provides an effective treatment for severe PGD after heart transplantation, which can promote graft function recovery.

## Introduction

At present, orthotopic heart transplantation is the most effective treatment for patients with end-stage heart disease. According to the 36th Annual Adult Heart Transplantation Registry report published by the International Society of Heart and Lung Transplantation (ISHLT) registry in 2019, a total of 1,31,249 adult heart transplants were completed by June 30, 2018, although >90% of the ISHLT data came from North America and Europe (1). The perioperative success rate of heart transplantation is about 90%. Shortage of donor hearts and time of donor heart ischemia are the main limitations restricting the development of heart transplantation (2).

Early primary graft dysfunction (PGD) after heart transplantation, especially right heart failure, is an important factor leading to perioperative death, affecting 7.4–36% of heart transplant recipients (3–5). The 30-day all-cause mortality for PGD has been reported to be about 19–30% (3, 4, 6). Although advances have been achieved in the field of transplantation in the past few decades, factors leading to PGD and associated treatment remain unclear (7). ISHLT has proposed a consensus definition for PGD in 2014, which standardized and graded its diagnosis. Inotropes can be used for mild to moderate PGD to restore myocardial contractility and maintain hemodynamic stability, including catecholamines, phosphodiesterase inhibitors, and levosimendan or intraaortic balloon counterpulsation (IABP). However, for patients with severe PGD, veno-arterial extracorporeal membrane oxygenation (VA-ECMO) is needed to maintain hemodynamic stability and perfusion of vital organs (3).

Veno-arterial extracorporeal membrane oxygenation is a temporary cardiopulmonary support technology that can provide effective circulation for critically ill patients with heart failure caused by a variety of reasons (8). In heart transplantation, VA-ECMO is mainly used in patients waiting for donor hearts before the operation and in patients with cardiogenic shock or PGD after the surgery. The survival rate in patients on VA-ECMO support is notably lower, especially in the early posttransplantation period. The Zhongshan Hospital Affiliated with Fudan University started to offer ECMO support therapy in 2009 and has achieved favorable therapeutic effects. This study aimed to investigate the timing and outcomes of VA-ECMO in patients with severe PGD after heart transplantation according to the ISHLT criteria.

## Materials and Methods

### Patients

This retrospective single-center study reviewed the records of 130 adult patients who underwent orthotopic heart transplantation at Zhongshan Hospital, Fudan University (Shanghai, China) between January 2014 and December 2020. According to the ISHLT criteria, patients with severe PGD who received VA-ECMO support within 24 h after heart transplantation surgery were included in this study. Exclusion criteria included age of <18 years and/or pregnancy. The study was approved by the Ethics Committee of Zhongshan Hospital. Patients were divided into two groups according to whether successful weaning from VA-ECMO occurred (patients were defined as successfully weaned from VA-ECMO if they survived for longer than 48 h after VA-ECMO explantation) (9, 10). Baseline variables, including age, sex, body mass index, comorbidities, laboratory tests, and heart failure etiology, together with outcome variables, which included VA-ECMO support time, VA-ECMO weaning rate, mechanical ventilation time, length of stay in intensive care unit (ICU), length of hospital stay, complications, and in-hospital mortality, were compared between the two groups. Preoperative laboratory tests refer to the tests made on the day or the day before the heart transplantation. The changes in the Sequential Organ Failure Assessment (SOFA) score and lactate (Lac) level from baseline to day 5 were retrospectively analyzed. All data were collected from the patients' hospital records by two residents (YJ-Z and JY-H).

### Surgical Procedures

After successful anesthesia, median sternotomy was performed to establish cardiopulmonary bypass (CPB). The superior vena cava (SVC), inferior vena cava (IVC), and ascending artery were blocked. The main trunk of the aorta and pulmonary artery, SVC, and IVC was cut off. The donor heart was pruned, and the posterior wall of the left atrium between the donor heart and the recipient heart was sutured continuously. The donor heart and recipient aorta were also sutured continuously, followed by aortic crossclamp removal to regain rhythmic contractions in the graft. The anastomosis with IVC, pulmonary artery, and SVC was accomplished in the beating heart to reduce the ischemia time of donor hearts.

### Definition of PGD

The specific diagnostic criteria were based on the 2013 ISHLT consensus (3). The PGD diagnosis was based on the evidence of cardiac dysfunction in the first 24 h after heart transplantation, including left, right, or total heart dysfunction. The clinical manifestation included severe hemodynamic instability with cardiogenic shock, excluding acute graft failure caused by other reasons, such as pericardial tamponade and hyperacute rejection. Although the ISHLT consensus committee recommended a distinction between PGD-LV and PGD-RV, it was not feasible to include it in this study because left and right ventricular assist devices (LVADs and RVADs) were not available in our center. VA-ECMO has become the preferred treatment for patients with refractory cardiogenic shock.

### Indications for VA-ECMO

The decision to use VA-ECMO was made by the cardiac surgeon in the operating room or by the intensivist in the cardiac surgery ICU. Indications for VA-ECMO therapy included difficulty weaning from CPB or postoperative refractory cardiogenic shock despite adequate volumes and high doses of inotropes, such as norepinephrine, dobutamine, epinephrine, and milrinone. A femoral venous cannula placed from the femoral vein to the right atrium was used as the VA-ECMO venous cannula. The femoral artery is most commonly used for arterial catheterization in adult patients. When femoral ECMO was initiated, an additional 8-Fr cannula was inserted distally into the femoral artery to prevent lower extremity ischemia.

### General Management During VA-ECMO Support

The optimal management of VA-ECMO involves several aspects, including circulatory support, anticoagulation, infection prevention, and nutritional support (11). The VA-ECMO support management protocol has been previously described (12). Briefly, the VA-ECMO blood flow and vasoactive drug dose were adjusted to maintain the mean arterial pressure above 65 mmHg. Several indices were used for the assessment of peripheral perfusion in VA-ECMO patients, such as clinical assessment (urinary output, skin mottling, capillary refill time, and consciousness), lactate level, mixed venous oxygen saturation, central venous oxygen saturation, and regional saturation of tissue oxygen. Cardiac function and hemodynamic conditions were routinely assessed by transesophageal or transthoracic echocardiography. Heparinization therapy was titrated according to the activated clotting time (ACT) and activated partial thromboplastin time (APTT). The target ACT was maintained at 180–200 s and APTT at 50–70 s (13). Platelets were transfused when the patient's platelet count fell below 50 × 10^9^/L (14). Major bleeding was defined if there was clinically overt bleeding recorded in the medical and/or nursing charts associated with either administration of 2 or more RBC units in 24 h or a drop in hemoglobin >2 g/L over 24 h, or if there was a hemothorax, central nervous system, or retroperitoneal bleeding, or if bleeding required surgical intervention. Midazolam and remifentanil were used for sedation.

### Weaning Protocol

When the patient showed signs of partial circulatory recovery and the echocardiographic evaluation demonstrated improvement in ventricular contractility, a VA-ECMO weaning test was performed by gradually reducing the pump flow to 1.5–2 L/min (15). In this setting, the removal of VA-ECMO support was considered if the patient's hemodynamic status remained stable, the left ventricular ejection fraction (LVEF) was ≥20–25%, the left ventricular outflow tract velocity time integral was ≥10 cm, and tissue Doppler lateral mitral annulus peak systolic velocity was ≥6 cm/s under minimal VA-ECMO support (16). Epinephrine was routinely used during the weaning process. In addition, when VA-ECMO combined with continuous renal replacement therapy (CRRT) or IABP was used, VA-ECMO could be removed first, and CRRT or IABP could be removed after the condition was further improved.

### Statistical Analysis

Continuous data were expressed as median (IQR). Categorical variables were expressed as percentages (%). Quantitative variables were analyzed using the Mann–Whitney U test. Categorical variables were analyzed by the chi-squared test or Fisher's exact methods as appropriate. Log-rank testing and Kaplan–Meier survival curves were used for survival analysis. Statistical significance was defined as *p* < 0.05. All statistical analyses were performed using the SPSS software (version 20.0; SPSS, Inc., Chicago, IL, USA).

## Results

**Figure 1:**
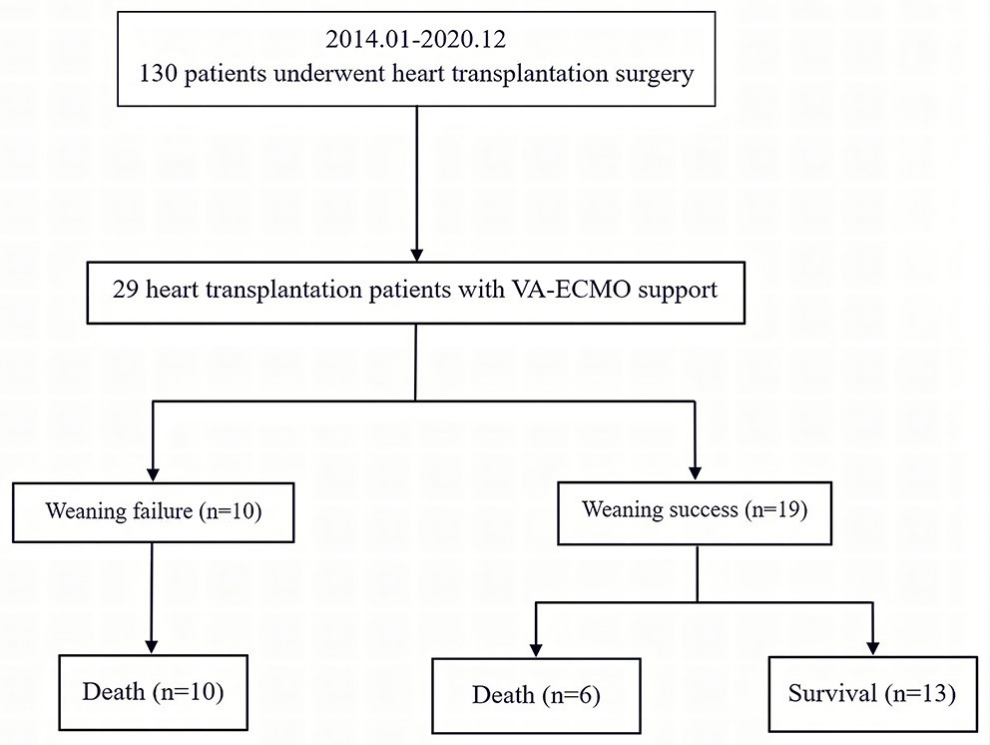
Enrollment, allocation, and follow-up for heart transplant patients who received VA-ECMO. VA-ECMO, veno-arterial extracorporeal membrane oxygenation.

A total of 130 patients underwent an orthotopic heart transplantation procedure between January 2014 and December 2020. Twenty-nine patients who were supported with VA-ECMO following heart transplantation surgery were enrolled in the study (Figure 1). None of the patients received mechanical circulatory support preoperatively. The overall incidence of severe PGD was 22.3%. The mean recipient age was 47 ± 14 years and 23 (79%) of the recipients were men. All of the patients had a pulmonary artery catheter inserted preoperatively. Demographics, comorbidities, laboratory tests, and clinical manifestations in the successful (*n* = 19) and failed (*n* = 10) weaning groups are summarized in Table 1. Most variables were similar between the two groups. However, patients in the successful group were likely to have a lower EuroSCORE (7 ± 2 vs. 10 ± 3; *p* < 0.01) compared to those in the failure group.

**Table 1:** Demographic and clinical characteristics of ECMO patients prior to surgery.

**Variables**	**Total** **(***n*** = 29)**	**Wean-from ECMO**		***p*** **-value**
		**Success** **(***n*** = 19)**	**Failure** **(***n*** = 10)**	
Age, *y*	48 (36,59)	54 (39,58)	41 (32,64)	0.77
Male, *n* (%)	23 (79)	17 (89)	6 (60)	0.14
BMI, (kg/m^2^)	23.03 (21.0,25.61)	23.03 (20.76,25.96)	21.84 (21.14,23.77)	0.46
**Comorbidities**, ***n*** **(%)**				
Hypertension	3 (10)	2 (11)	1 (10)	1.00
Diabetes mellitus	2 (7)	1 (5)	1 (10)	0.96
CKD	3 (10)	1(5)	2 (20)	0.27
Atrial fibrillation	3 (10)	2 (11)	1 (10)	1.00
Pulmonary hypertension	7 (24)	4 (21)	3 (30)	0.66
**Diagnosis**				
Ischemic heart disease	2 (7)	2 (11)	0 (0)	0.53
Dilated cardiomyopathy	19 (66)	14 (73)	5(50)	0.24
Congenital heart disease	4 (14)	1 (5)	3 (30)	0.10
Valvular heart disease	4 (14)	2 (11)	2 (20)	0.59
Previous cardiac surgery, n (%)	8 (28)	3 (16)	5 (50)	0.08
Preoperative PVR, (wood units)	2.9 (2.2,4.5)	3.3 (2.0,4.4)	2.8 (2.2,5.2)	0.88
**Laboratory tests**				
cTnT, (ng/ml)	0.03 (0.02,0.07)	0.03 (0.02,0.06)	0.04 (0.01,0.09)	0.67
BNP, (pg/ml)	4890 (2406,9728)	4890 (2479,10930)	4273 (2168,9628)	0.51
Hb, (g/L)	137 (124,147)	139 (128,147)	129 (95,146)	0.33
Hct, (%)	40.6 (35.1,44.3)	41.2 (39.4,44.2)	37.4 (31.3,44.5)	0.31
WBC, (× 10^12^/L)	6.94 (4.99,8.47)	6.81 (4.89,8.34)	7.22 (5.45,9.21)	0.70
Neutrophils, (%)	69.2 (59.6,73.6)	68.8 (57.9,73.0)	69.8 (62.2,75.6)	0.54
PLT, (× 10^9^/L)	182 (152,223)	176 (134,222)	206 (159,238)	0.23
ALB, (g/L)	43 (37,46)	42 (35,46)	43 (38,46)	0.95
TBIL, (μmol/L)	27 (19,47)	28 (18,44)	27 (22,51)	0.74
DBIL, (μmol/L)	10 (6,21)	9 (6,20)	16 (8,24)	0.43
ALT, (U/L)	28 (18,43)	30 (19,46)	25 (12,29)	0.18
AST, (U/L)	30 (25,38)	37 (26,41)	30 (22,35)	0.40
Cr, (μmol/L)	101 (82,118)	101 (81,118)	107 (77,127)	0.80
BUN, (mmol/L)	9 (7,11)	9 (8,11)	9 (6,12)	0.80
CRP, (mg/L)	2.3 (1.5,11.7)	2.3 (1.5,10.5)	2.2 (1.5,14.2)	0.91
T3, (nmol/L)	1.3 (1.1,1.5)	1.3 (1.0,1.5)	1.3 (1.1,1.5)	0.94
T4, (nmol/L)	98.4 (85.7,105.9)	96.5 (82.6,107.0)	99.7 (93.7,106.8)	0.60
TSH, (uIU/mL)	3.54 (2.15,5.58)	3.54 (2.16,5.69)	3.02 (2.13,6.02)	1.0
PT, (s)	16.5 (13.3,19.3)	15.8 (12.2,20.1)	18.0 (16.1,19.0)	0.25
INR	1.4 (1.1,1.7)	1.4 (1.1,1.8)	1.5 (1.4,1.7)	0.31
APTT, (s)	32.7 (27.2,36.7)	31.3 (26.9,35.2)	33.8 (28.1,40.5)	0.18
Fib, (mg/dL)	264 (221,333)	264 (208,332)	276 (222,349)	0.70
EuroSCORE	8 (6,11)	7 (5,9)	10 (9,12)	0.01
APACHE II	14 (7,22)	13 (7,18)	20 (7,28)	0.14
LVEF, (%)	28 (25,35)	27 (25,29)	32 (24,41)	0.33

*Continuous data are presented as the mean (SD) or median (IQR). Categorical data are presented as counts (%)*.

*ECMO, extracorporeal membrane oxygenation; BMI, body mass index; cTnT, cardiac troponin T; BNP, brain natriuretic peptide; Hb, hemoglobin; PLT, platelet; TBIL, total bilirubin; DBIL, direct bilirubin; ALT, alanine aminotransferase; AST, aspartate aminotransferase; Cr, serum creatinine; BUN, blood urea nitrogen; CRP, C-reactive protein; TSH, thyroid stimulating hormone; PT, prothrombin time; INR, international normalized radio; APTT, activated partial thromboplastin time; Fib, fibrinogen; EuroSCORE, European System for Cardiac Operative Risk Evaluation; APACHE II, Acute Physiology and Chronic Health Evaluation; LVEF, left ventricular ejection fraction*.

Perioperative details of the 29 patients who were supported with VA-ECMO are shown in Table 2. Although there were no significant differences in operation, CPB, or graft ischemia durations between the two groups, the durations in the failed group were longer than those in the successful group (411 ± 81 vs. 484 ± 116 min; 236 ±65 vs. 280 ± 66 min; 246 ± 118 vs. 274 ± 129 min, *p* > 0.05). Postoperative peak alanine aminotransferase (ALT) and aspartate aminotransferase (AST) levels were both significantly higher in patients who failed to wean from VA-ECMO(39 [25–51] vs. 141 [51–806] U/L; 128 [84–151] vs. 275 [162-1,506] U/L, respectively; *p* < 0.01). Nineteen patients received VA-ECMO support in the operating room due to difficulty with weaning from CPB. Ten patients had VA-ECMO initiated in the cardiac surgery ICU because of refractory postoperative cardiogenic shock. The median time from the end of the operation until VA-ECMO implantation was 8.6 h in the ICU group.

**Table 2:** Intraoperative and postoperative clinical characteristics.

**Variables**	**Total** **(***n =*** 29)**	**Wean-from ECMO**		***p*** **-value**
		**Success (*n =* 19)**	**Failure (*n =* 10)**	
**Intraoperative conditions**				
Operation time (min)	420 (370,503)	420 (355,472)	470 (380,575)	0.15
CPB time (min)	245 (210,304)	230 (193,290)	275 (218,332)	0.12
Aortic cross clamp time (min)	53 (44,62)	54 (45,82)	51 (37,57)	0.27
Donor organ ischemic time (min)	285 (127,370)	230 (134,360)	342 (120,382)	0.57
**Post-ECMO support conditions**
Red blood cell transfusion (U)	14 (9,20)	15 (12,19)	11 (7,22)	0.20
Frozen plasma (ml)	2000 (900,3000)	2000 (1400,3000)	1700 (600,2800)	0.46
Drainage in first three days	1670 (1260,2320)	1590 (1270,2260)	2165 (1238,3085)	0.29
Peak cTnT (ng/ml)	2.61 (1.88,5.28)	2.16 (1.78,5.75)	3.58 (2.00,4.85)	0.70
Peak BNP (pg/ml)	15565 (8012,29527)	15565 (6495,25646)	14936 (8603,48817)	0.74
Peak lactate (mmol/L)	12.0 (10.4,19)	12.0 (9.0,15.5)	15.4 (11.7,20)	0.13
Peak TBIL (μmol/L)	76.5 (46.2,93.1)	75.6 (39.9,92)	85.0 (58.3,140.7)	0.38
Peak DBIL (μmol/L)	51.0 (26.1,69.5)	40.0 (25.3,67.6)	60.7 (24.1,124.4)	0.27
Peak ALT (U/L)	51 (27,137)	39 (25,57)	141 (51,806)	0.01
Peak AST (U/L)	136 (92,275)	128 (84,151)	275 (162,1506)	0.01
Peak Cr (mmol/L)	228 (193,301)	221 (182,295)	247 (216,336)	0.46

*ECMO, extracorporeal membrane oxygenation; CPB, cardiopulmonary bypass; cTnT, cardiac troponin T; BNP, brain natriuretic peptide; TBIL, total bilirubin; DBIL, direct bilirubin; ALT, alanine aminotransferase; AST, aspartate aminotransferase; Cr, serum creatinine*.

**Figure 2:**
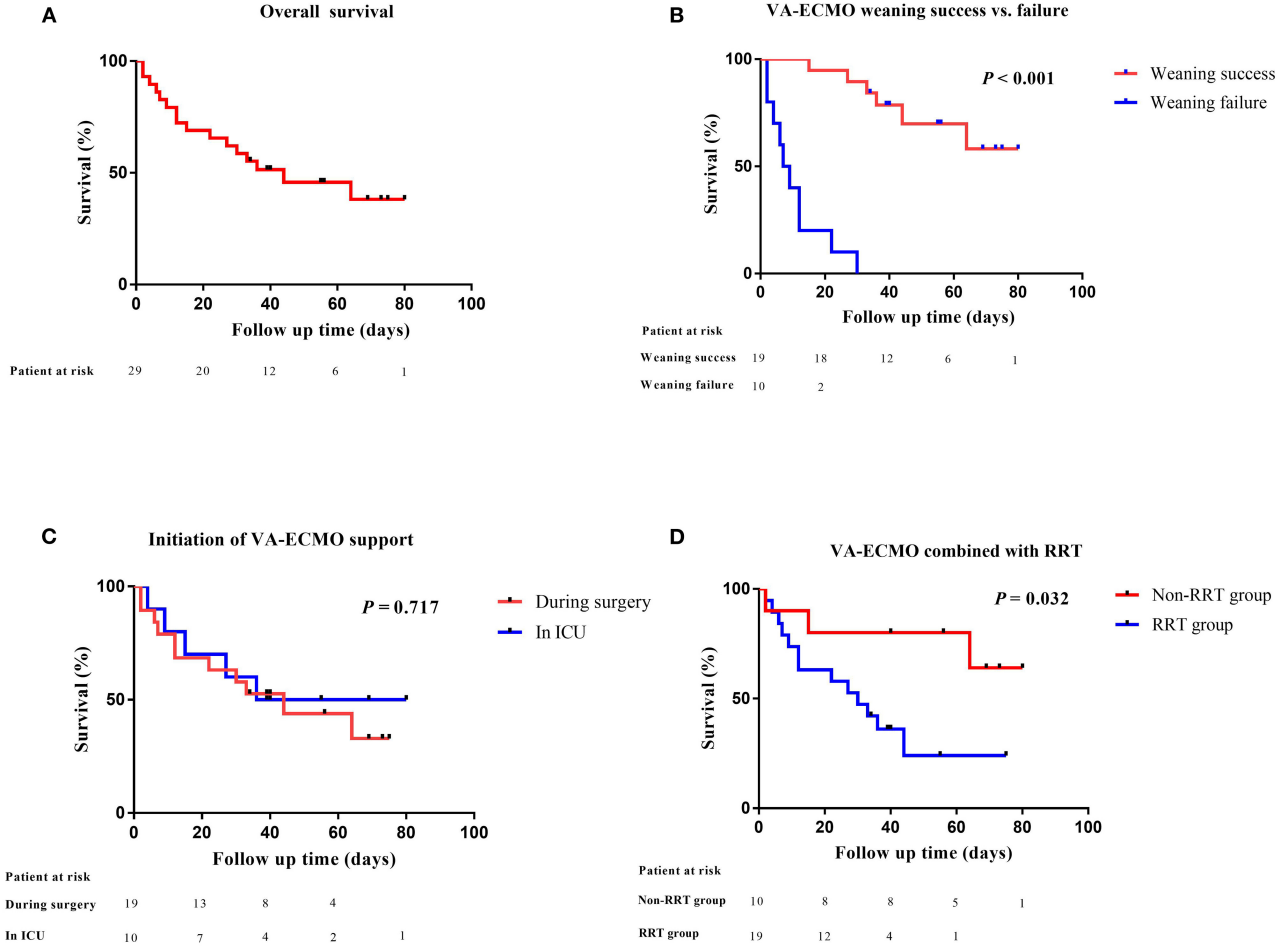
Comparative survival in heart transplant patients after VA-ECMO support. **(A)** Kaplan–Meier analysis of overall survival in heart transplant patients supported by VA-ECMO from 2014 to 2020 (*n* = 29); **(B)** VA-ECMO weaning success or failure; **(C)** initiation of VA-ECMO support; **(D)** VA-ECMO combined with RRT. VA-ECMO, veno-arterial extracorporeal membrane oxygenation; ICU, intensive care unit; RRT, renal replacement therapy.

**Figure 3:**
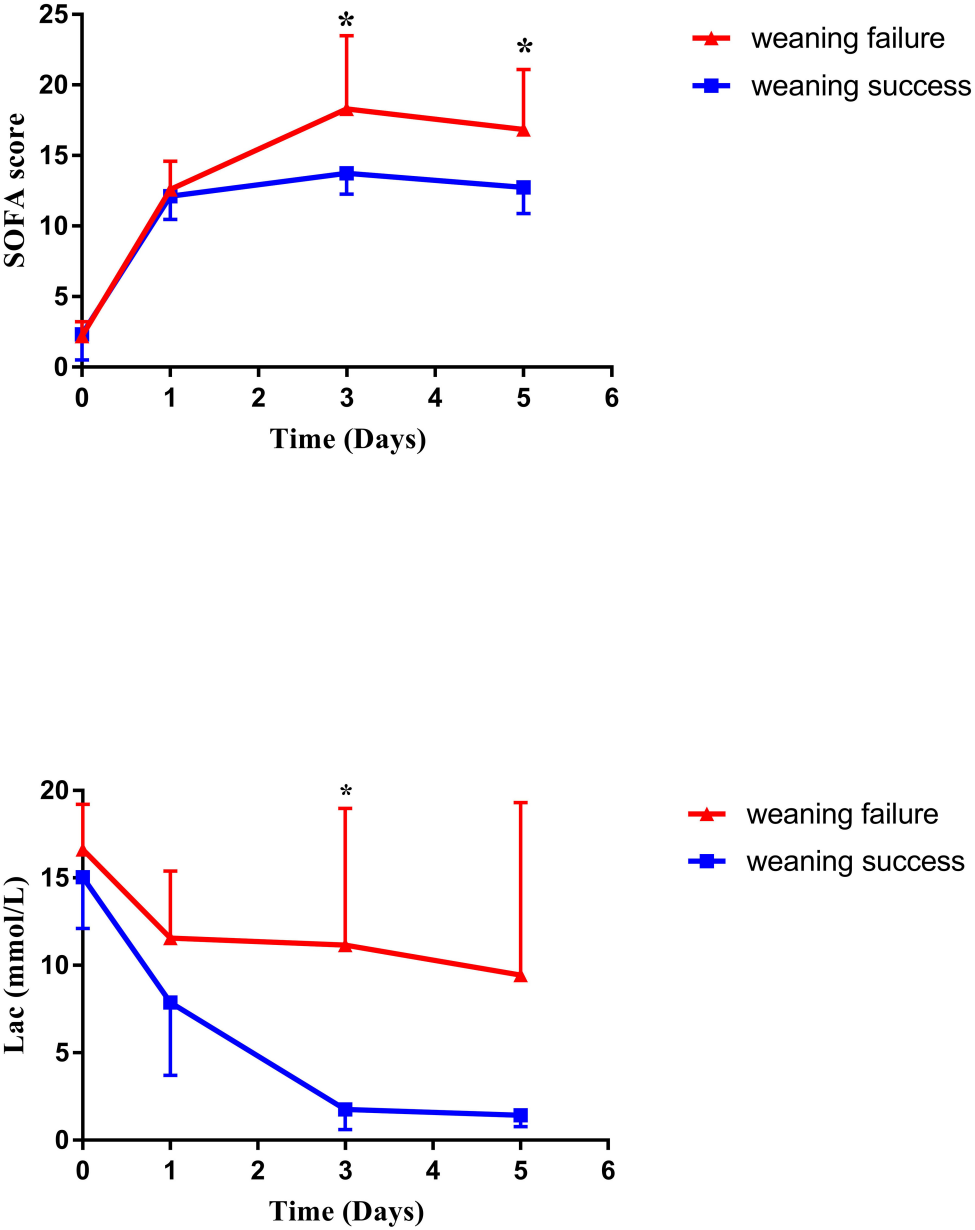
Changes in SOFA score and lactate level during VA-ECMO support. SOFA, sequential organ failure assessment; VA-ECMO, veno-arterial extracorporeal membrane oxygenation.

After a median support period of 5 days, 19 patients (66%) were successfully weaned from VA-ECMO support, whereas six patients died in the hospital despite successful weaning from VA-ECMO. Thirteen patients (45%) survived until hospital discharge. No patient developed leg ischemia or leg fasciotomy due to femoral arterial cannulation. The Kaplan–Meier survival curves for patients with severe PGD who required perioperative VA-ECMO support are shown in Figure 2. SOFA scores were significantly higher in the weaning failure group on days 3 and 5, whereas the difference in Lac level was significant only on day 3 (Figure 3).

Additionally, six of the patients who were successfully weaned from VA-ECMO died, including four patients died of infection or sepsis (21, 59, 8, and 30 days after weaning), one patient died of ventricular arrhythmia (31 days after weaning), and one patient died of neurological complications (19 days after weaning). No patient was lost to follow-up during this period. In addition, VA-ECMO treatment of severe PGD had no adverse effect on graft function among survivors. Details for VA-ECMO implementation and outcomes in this patient are outlined in Table 3.

**Table 3:** ECMO implementation and clinical outcomes.

**Variables**	**Total** **(***n =*** 29)**	**Wean-from ECMO**		***p*** **-value**
		**Success** **(***n =*** 19)**	**Failure** **(***n =*** 10)**	
Initiation of ECMO support (*n*)				0.41
During surgery	19 (66)	11 (58)	8 (80)	
In ICU	10 (34)	8 (42)	2 (20)	
ECMO duration (*d*)	5 (3,7)	5 (5,7)	5 (1,7)	0.38
MV time (*d*)	10 (7,18)	13 (9,19)	6 (2,11)	0.01
CRRT, *n* (%)	19 (66)	10 (53)	9 (90)	0.10
IABP, *n* (%)	4 (14)	1 (5)	3 (30)	0.10
**Cause of death**, ***n*** **(%)**				
Major bleeding	3 (10)	0 (0)	3 (30)	0.01
Infection/sepsis	4 (14)	4 (21)	0 (0)	0.27
VT/VF	3 (10)	1 (5)	2 (20)	0.10
Neurological complications	2 (7)	1 (5)	1 (10)	0.27
Graft failure	4 (14)	0 (0)	4 (40)	0.01
ICU stay (*d*)	20 (10,29)	24 (17,38)	6 (2,11)	0.01
Hospital stay (*d*)	36 (11,56)	40 (36,69)	8 (4,12)	0.01
In-hospital mortality, *n* (%)	16 (55)	6 (32)	10 (100)	0.01

*ECMO, extracorporeal membrane oxygenation; ICU, intensive care unit; MV, mechanical ventilation; CRRT, continuous renal replacement therapy; IABP, intra aortic balloon counterpulsation; VT, ventricular tachycardia; VF, ventricular fibrillation*.

## Discussion

In this single-center study, 29 out of 130 heart transplant recipients received VA-ECMO and achieved relatively satisfactory results. Because LVADs and RVADs were not available in our center, VA-ECMO was the only effective treatment for patients with severe PGD after heart transplantation. To the best of our knowledge, this study is the first to apply the new ISHLT criteria to Chinese patients to investigate outcomes of severe PGD at high-volume transplantation centers.

Over the last several decades, orthotopic heart transplantation has become the standard treatment for select patients with advanced and refractory heart failure. However, severe PGD remains a major cause of perioperative death after heart transplantation. Several studies have investigated the incidence of severe PGD using the ISHLT consensus statement criteria and found that the incidence rate was 7–23% with in-hospital mortality of 19–65% (4, 6, 17–21). VA-ECMO is a viable extracorporeal life support technique for cardiac surgery patients who are difficult to wean from CPB or those with postoperative cardiogenic shock. In this study, the successful weaning rate and mortality following VA-ECMO were 66 and 55%, respectively, which are comparable to those published by Alessandro et al. (weaning: 60%, survival: 46%) (20). We believe that the main causes of this higher incidence rate and mortality may be associated with marginal donors, basic patient conditions, surgical strategies, longer cold ischemia duration, and indication for VA-ECMO despite its likelihood to be multifactorial (4, 5, 22).

The timing of VA-ECMO implantation is an important factor influencing patient outcomes. A previous study has shown that prompt implementation of VA-ECMO allows for adequate organ perfusion and promotes the recovery of graft function, while avoiding multiple organ dysfunction and complications caused by large doses of inotropic or vasoactive agents (17). This study compared outcomes in patients with different VA-ECMO initiation times and found no significant differences between the two groups in terms of weaning success rate or in-hospital mortality. It should be noted that the median time from the end of the operation to the start of VA-ECMO was 8.6 h in the ICU group. It has been shown that delayed VA-ECMO initiation for longer than 24 h in heart transplant recipients with refractory cardiogenic shock can lead to poor outcomes (6, 17, 23). Based on our previous experience, we were more aggressive in using VA-ECMO as a therapy for severe PGD in the ICU.

It has been reported that successful weaning from VA-ECMO does not mean patient survival. Several studies have assessed the predictors of death after VA-ECMO weaning in postcardiotomy shock patients. VA-ECMO implantation time, poor renal and liver function, high lactate levels, and high SOFA scores were reported to be predictors of death after weaning (24–26), which is similar to the results in this study. Many scoring systems are routinely used in cardiac surgery ICU. SOFA was developed to objectively evaluate the degree of severity in ICU patients and to simplify the prediction of patients' risk of mortality (27). Nevertheless, in patients with VA-ECMO support, the performance of the SOFA score in predicting short-term mortality is still controversial (25, 28).

Complications during VA-ECMO support may directly lead to recipient's death or forced weaning from VA-ECMO. The leading causes of death in this study were surgical bleeding, graft failure, and septic shock. The causes of major postoperative bleeding are multifactorial (29–33). Excessive loss of coagulation factors during operation, longer CPB time, thrombocytopenia, massive perioperative blood transfusion, and postoperative VA-ECMO support can lead to coagulation dysfunction and relentless bleeding. Three patients died of major bleeding in this study. Therefore, it is very important to strictly maintain hemostasis during the operation, provide infusion of fresh frozen plasma, platelets, fibrinogen, and prothrombin complex, closely observe drainage after the operation, and conduct a secondary thoracotomy if necessary. How to best maintain the balance between bleeding and clotting in VA-ECMO patients remains a challenge.

There were several limitations in this study. First, this was a single-center retrospective study. Second, although the analyses were based on the experience over the past 7 years, the sample size was too small and the follow-up time was relatively short, which meant that detailed risk factor analysis was not possible. Third, due to privacy protection, we are unable to obtain donor information. In the future, multicenter studies with large patient populations are needed to optimize management strategies and to improve outcomes in this rare but complex cardiac emergency.

## Conclusion

In conclusion, the application of VA-ECMO in the perioperative period of heart transplantation provided a “bridge to recovery” in patients with severe PGD. Further research is needed to determine the optimal time for VA-ECMO use and how to prevent complications to improve patient prognosis.

## Data Availability Statement

The original contributions presented in the study are included in the article/supplementary materials, further inquiries can be directed to the corresponding author/s.

## Ethics Statement

The studies involving human participants were reviewed and approved by the Ethics Committee of Zhongshan Hospital affiliated to Fudan University (No. B2020-169). The patients/participants provided their written informed consent to participate in this study.

## Author Contributions

J-yH, G-wT, and ZL contributed to study design. XL, Y-jZ, J-lZ, and J-fM contributed to participant enrollment. K-fG and S-gY contributed to study management and data collection. J-yH and S-gY contributed to manuscript writing. G-wT and ZL contributed to data analyses and manuscript revision. All authors have read and approved the final manuscript.

## Funding

This study was supported by grants from the Natural Science Foundation of Shanghai (20ZR1411100 to ZL, 21ZR1412900 to YS), the Science and Technology Commission of Shanghai Municipality (20DZ2261200 to ZL), the National Natural Science Foundation of China (82070085 to G-wT), the Construction Program of Key but Weak Disciplines of Shanghai Health Commission (2019ZB0105 to ZL), the Clinical Research Project of Zhongshan Hospital (2020ZSLC38 to G-wT, 2020ZSLC27 to ZL), the Smart Medical Care of Zhongshan Hospital (2020ZHZS01 to ZL), the Project for Elite Backbone of Zhongshan Hospital (2021ZSGG06 to G-wT), and the Foundation for Young Researchers of Zhongshan Hospital (2021ZSQN22, 2021ZSQN71, and 2021ZSQN72).

## Conflict of Interest

The authors declare that the research was conducted in the absence of any commercial or financial relationships that could be construed as a potential conflict of interest.

## Publisher's Note

All claims expressed in this article are solely those of the authors and do not necessarily represent those of their affiliated organizations, or those of the publisher, the editors and the reviewers. Any product that may be evaluated in this article, or claim that may be made by its manufacturer, is not guaranteed or endorsed by the publisher.
